# Morphological and Molecular Studies of Tetracotyle-Type Metacercariae of the Genus *Cotylurus* Szidat, 1928 (Trematoda) from the Gravel Snail *Lithoglyphus naticoides* (Gastropoda) and Host Sex Dependent Differences in Infection Rate

**DOI:** 10.3390/pathogens14101063

**Published:** 2025-10-20

**Authors:** Gražina Stanevičiūtė, Virmantas Stunžėnas, Romualda Petkevičiūtė

**Affiliations:** State Scientific Research Institute Nature Research Centre, Akademijos 2, LT-08412 Vilnius, Lithuania; grazina.staneviciute@gamtc.lt (G.S.); romualda.petkeviciute@gamtc.lt (R.P.)

**Keywords:** tetracotyle, *Cotylurus* spp., ITS2 rDNA, 28S, molecular phylogeny, host sex dependent infection

## Abstract

The Ponto-Caspian invader, the gravel snail *Lithoglyphus naticoides* (C. Pfeiffer, 1828), is infected with a diverse community of digenetic trematodes in its colonized range and most often serves as first intermediate host. We have performed the parasitological examination of *L. naticoides* snails sampled in Kaunas water reservoir (Lithuania) and found yet unknown strigeid metacercariae of the tetracotyle type using these snails as second intermediate host. In this study, we report data on morphology and molecular analysis based on two markers, the partial 28S rDNA gene and the ITS2 region of these metacercariae. Based on the comparative molecular and phylogenetic analysis, the metacercaria detected in *L. naticoides* was identified as *Cotylurus cornutus* (Rudolphi 1809) Szidat, 1928. Differences in metacercariae infection between snail sexes were assessed. The prevalence of infection in *L. naticoides* was significantly higher in males than in females. Additional molecular markers of tetracotyle of *C. cornutus* from *Stagnicola palustris* (O. F. Müller, 1774) and furcocercaria of *Cotylurus strigeoides* Dubois, 1958 from *Physa fontinalis* (Linnaeus, 1758), sampled in Curonian Lagoon, Lithuania, were obtained for the first time and used for comparative phylogenetic analysis.

## 1. Introduction

Despite a long-lasting history of taxonomic studies on trematodes in Europe, our knowledge of species diversity of even widespread trematode species is incomplete, and their life-cycle stages are not linked to each other. Freshwater gastropod *Lithoglyphus naticoides* (C. Pfeiffer, 1828) is a non-indigenous species in Lithuanian inland waters. Originating from the Ponto-Caspian region, *L. naticoides* probably invaded the inland waters of Lithuania at the end of the 18th century [1,2] and the first published observations were in the Nemunas and Nevėžis Rivers in 1930s [3,4]. In the Nemunas River, especially in the lower reaches, this snail was among the most dominant species in the macroinvertebrate assemblage, indicating that under favorable environmental conditions it may attain high densities [5]. The negative ecological impacts of invasive species on ecosystems can be associated with species of parasites, introduced together with their host. *Lithoglyphus naticoides* is known as the first intermediate host of several trematode species [6,7,8,9,10,11], some of which are highly pathogenic to fish and fish-eating birds and mammals. Therefore, parasitological studies of mollusks are relevant in order to assess threats to local ecosystems due to the possibility of the spread of new species of parasites to them.

During our long-term parasitological examination of *L. naticoides* in the population of Kaunas water reservoir (Lithuania), metacercariae of the tetracotyle type were collected. We have determined that tetracotyle-type metacercaria has been observed for the first time in *L. naticoides*. These tetracotyles have remained unidentified due to the limited discriminative morphological characters and limited availability of reliable comparative sequences of adult Strigeidae trematodes in GenBank. However, recent molecular studies of different life-cycle stages of *Cotylurus* spp. created the basis for further studies of biodiversity and phylogeny [12].

Our current study provides data on infection parameters and morphological description of tetracotyles parasitizing snails from the population of Kaunas water reservoir and Curonian Lagoon, Lithuania, along with molecular phylogenetic analysis based on two molecular markers, partial 28S rDNA and the ITS2 region, including new data from *Cotylurus* spp. parasitizing *L. naticoides*, *Stagnicola palustris* (O. F. Müller, 1774), and *Physa fontinalis* (Linnaeus, 1758).

*Lithoglyphus naticoides* is dioecious, but its sexual dimorphism is not clearly apparent [13,14]. Some parasites are known to favor one sex over the other in their host species [15,16]. Given that this phenomenon is generally understudied, this study was the first to take host sex into account when assessing infection of *L. naticoides* with trematode larvae.

## 2. Material and Methods

A total of 780 *L. naticoides* specimens were collected by hand-net in Kaunas water reservoir, from August 2010 until October 2019; 10 specimens of *Stagnicola palustris* and 5 specimens of *P. fontinalis* were collected in Curonian Lagoon in September 2024. In the laboratory, the snails were dissected using a stereomicroscope MBS-9 (LOMO, St. Petersburg, Russia) and sexed based on the morphology of the reproductive organs [13,14]. The males were identified during dissection: after cutting off part of the shell and carefully removing the soft tissues of the mollusk from the remaining part of the shell, the male’s penis, situated posteriorly to the right tentacle, was easily visible. Visceral organs were squeezed between two glass plates and examined for the presence of metacercariae. Encysted, not stained metacercariae were studied alive, and photographs were taken with a Moticam Pro 252A camera (Motic China Group Co., Ltd., Xiamen, China), mounted on the light microscope Olympus BX51 (Olympus Corporation, Tokyo, Japan) for measurement and further identification.

The tetracotyle metacercariae developmental stages are divided into three general age groups [17,18]. These include “developing” forms—all stages up to the largest in size and those beginning to undergo reorganization of the body—involving a breakdown of almost all internal organs (the suckers disappearing entirely, translucent body, increasingly large amount of granular material obscuring details of internal morphology), “pre-cysts” (those in which a hind-body has developed but which do not yet possess a cyst wall), and “cysts” (fully formed tetracotyles surrounded by a distinct cyst wall).

### Molecular and Phylogenetic Analyses

The collected larvae specimens were fixed in 96% ethanol. Genomic DNA for molecular analysis was extracted from ethanol-fixed tetracotyles following the Stunžėnas’ protocol [18,19]. Amplification and sequencing of the two rDNA markers, the nuclear internal transcribed spacers (ITS1-5.8S-ITS2 rDNA) and the beginning of the 28S ribosomal gene-coding regions, were performed following the protocol used in our previous studies [20]. A fragment at the 5′ end of the 28S rRNA gene was amplified using the forward primers Digl2 (5′-AAG CAT ATC ACT AAG CGG3-′) or ZX-1 (5′-ACC CGC TGA ATT TAA GCA TAT3-′) [21] and the reverse primers L0 (5′-GCT ATC CTG AG (AG) GAA ACT TCG3-′) [22] or 1500R (5′-GCT ATC CTG AGG GAA ACT TCG3-′) [23,24].

Polymerase chain reaction (PCR) products were purified and sequenced by the Sanger sequencing method in both directions at Macrogen Europe (Amsterdam, The Netherlands) using the PCR primers. Contiguous sequences were assembled using Sequencher 4.10.1 software (Gene Codes Corporation, Ann Arbor, MI, USA). Mean evolutionary divergence over sequence pairs within and between groups was estimated using MEGA v.11.0.11 software [25]. Newly generated sequences were compared with identical, similar and related sequences for phylogenetic analyses found by the “Basic Local Alignment Search Tool” (NCBI, Bethesd, MD, USA) on NCBI BLAST homepage (https://blast.ncbi.nlm.nih.gov/) [26]. Both the ITS2 and 28S datasets were aligned independently using ClustalW [27], integrated into MEGA v.11.0.11, with an open gap penalty of 15 and gap extension penalty of 6.66. The Maximum Likelihood (ML) trees were obtained using the general time reversible model with a gamma distribution rate (GTR + G) for both the ITS2 and the 28S gene datasets. The value for gamma and the number of invariant sites was estimated from the data. Parsimony analysis based on subtree pruning and regrafting (SPR) was used with default parsimony settings. Branch support was estimated by bootstrap analyses with 1000 pseudoreplicates. Furthermore, additional rDNA sequences of *Cotylurus* species were downloaded from GenBank and included in phylogenetic analysis (Table 1). Sequences of the *Alaria americana* Hall & Wigdor, 1918 and *Cardiocephaloides longicollis* (Rudolphi, 1819) were used as the outgroup for ITS2 and 28S phylogenetic trees. Additionally, a sequence of *Diplostomum ardeae* Dubois, 1969 was used in the outgroup for ITS2 tree, and *Neodiplostomum vaucheri* Dubois, 1983 was used for 28S tree (Table 1).

## 3. Results

### 3.1. Infection Parameters

A total of 780 *L. naticoides* specimens were examined; in 611 of them, the sex of the mollusk was determined during dissection. The balance of males and females in the *L. naticoides* population was approximately equal. Infection parameters are presented in Table 2 and Table 3. Larvae of tetracotyle type were found encysted in the digestive gland, gonads, or between the mantle and viscera of host snail. The observed prevalence of tetracotyle-type metacercariae detected in snails (19.7%) was relatively high. Males were more often infected than females. Our study carried out in 2010 found 42.6% infected males (26 infected from 61 ♂ dissected) and 6.6% infected females (4 from 61 ♀ dissected). Meanwhile, in 2011, during the dissection of 240 *L. naticoides* specimens, 45% of males (54 infected from 120 ♂ dissected) were found infected with tetracotyle larva, and only 3 females from 120 dissected ♀ (2.5%). During 2018, we dissected 207 *L. naticoides* (103 ♀, 104 ♂); 32 males (30.8%) and 8 females (7.8%) were infected with tetracotyle-type metacercariae. Intensity of infection varied from 1 to 15. During the study period, 2010–2019, we found 154 snails infected with 442 tetracotyle metacercariae; the mean intensity of infection was 2.9. The young larvae, “developing” forms according to the proposed classification of Cort et al. in 1941 [17], of tetracotyle metacercariae were found in samples collected in September of 2018.

### 3.2. Molecular Identification and Phylogenetic Analyses

The newly generated sequences were aligned and compared with sequences of *Cotylurus* specimens available in the GenBank (Table 1) that were not shorter than 1216 bp for the 28S alignment and not shorter than 410 bp for the ITS2 alignment. Alignments of the ITS2 and partial 28S datasets yielded 402 and 1216 characters for analysis, respectively.

**Figure 1:**
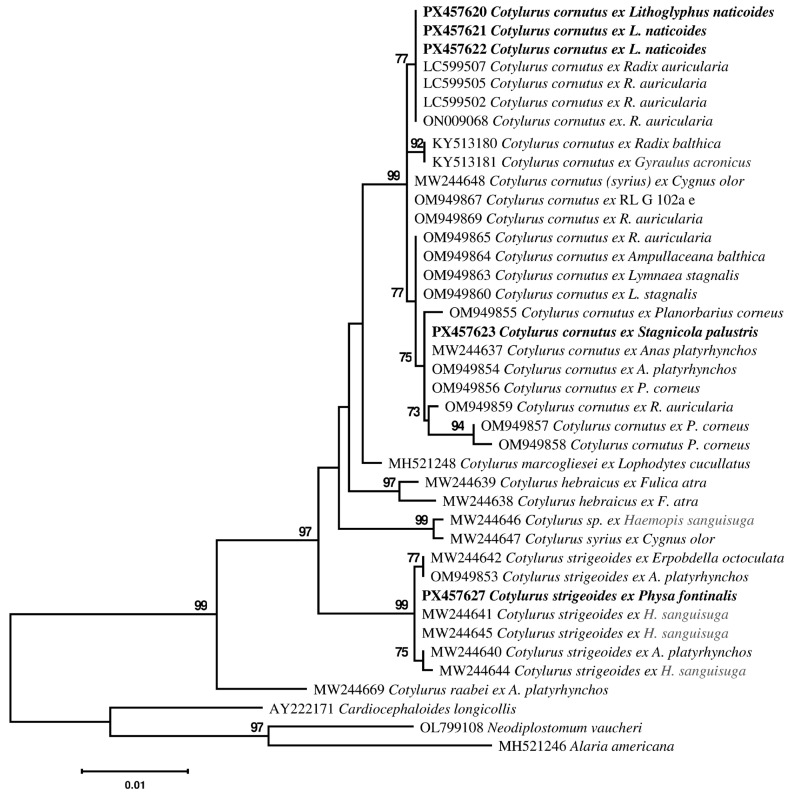
Phylogenetic tree based on Maximum Likelihood analysis of partial sequences of the 28S nuclear rDNA gene. Bootstrap support values lower than 70% are not shown. The Genbank numbers of sequences generated in this study are indicated in bold.

**Figure 2:**
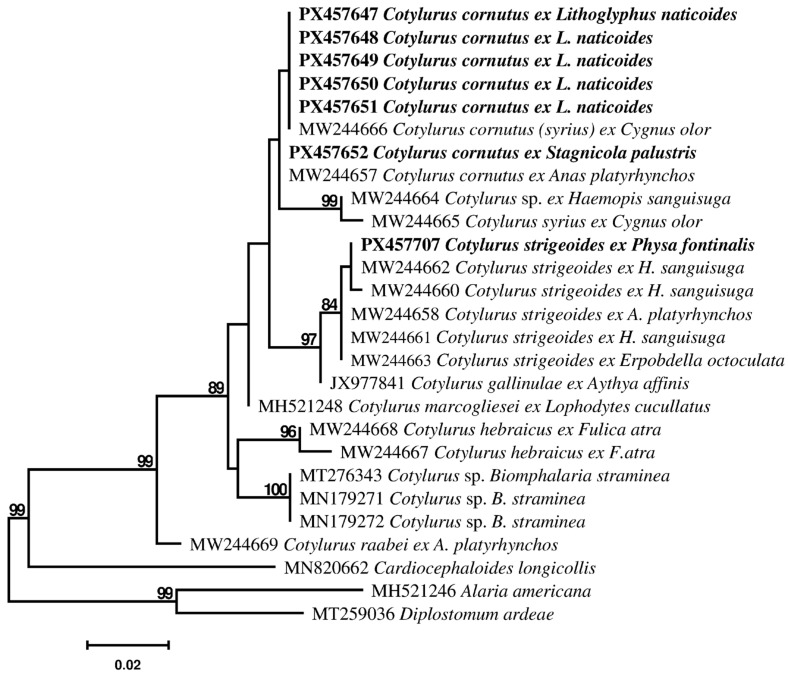
Phylogenetic tree based on Maximum Likelihood analysis of the ITS nuclear rDNA region. Bootstrap support values lower than 70% are not shown. The Genbank numbers of sequences generated in this study are indicated in bold.

Analyses of these datasets produced identical tree topologies (Figure 1 and Figure 2), despite the less available ITS2 sequences. All sequences of *Cotylurus* spp. and newly obtained sequences clustered there in one well-supported main monophyletic clade. The newly obtained sequences of tetracotyle metacercariae formed a 99% supported clade in 28S (Figure 1), together with sequences of *Cotylurus cornutus* (Rudolphi 1809) Szidat, 1928; their differences between sequences do not exceed more than 4bp (0.33%).

The 28S sequences of tetracotyle-type metacercariae from *S. palustris* appeared to be identical to sequences (MW244637, OM949854,) of *C. cornutus* from *Anas platyrhynchos* Linnaeus, 1758. These sequences of tetracotyle metacercariae from *S. palustris* have low divergence from the sequences of tetracotyle metacercariae from *L. naticoides*, 3 bp (0.25%) in the 28S dataset, 1bp (0.25%)-ITS2. Also, the 28S dataset comprises identical sequences (KY513180, KY513181) of *C. cornutus* from *Radix balthica* Linnaeus, 1758 and *Gyraulus acronicus* (A. Ferussac, 1807), they differ from the sequence of the adult *C. cornutus* by 4 bp, and they differ from the newly obtained sequences of tetracotyles found in *L. naticoides* and *S. palustris* by 4 bp.

In the 28S dataset, two different sequences of *Cotylurus syrius* Dubois, 1934 from *Cygnus olor* (J. F. Gmelin, 1789) were included, MW244648 and MW244647, their differences reached 17 bp (1.40%) in the interspecific level. The sequences of one of these specimens formed well-supported (99%) clades with 28S sequences (MW244646) of *Cotylurus* specimens from *Haemopis sanguisuga* (Linnaeus, 1858). The 28S sequences of other specimens, identified as *C. syrius*, were clustered in the *C. cornutus* clade and had only 1 bp difference from the 28S sequence of tetracotyle metacercariae from *L. naticoides.*

Almost identical differences were detected in ITS2 tree. In the ITS2 dataset, two different sequences of *C. syrius* from *C. olor* were included, MW244666 and MW244665, and their difference reached the interspecific level, 9 bp (2.24%). The ITS2 sequence (MW244665) of one of these specimens formed a well-supported (99%) clade with ITS2 sequences (MW244664) of *Cotylurus* specimens from *H. sanguisuga*. The ITS2 sequence (MW244666) of other specimens, identified as *C. syrius*, clustered in the *C. cornutus* clade and was identical with the ITS2 sequences of tetracotyle metacercariae from *L. naticoides.*

Sequences of furcocercariae from *P. fontinalis*, obtained for the first time, was clustered in a 99% supported clade in the 28S tree (Figure 1) and a 84% supported clade in the ITS2 tree (Figure 2) together with *C. strigeoides* Dubois, 1958 from *A. platyrhynchos*, and *Cotylurus* specimens from leeches *H. sanguisuga* and *Erpobdella octoculata* (Linnaeus, 1858)*;* their differences between sequences were intraspecific and did not exceed more than 3 bp (0.25%) in the 28S dataset and 1bp (0.25%) in the ITS2 dataset. The 28S and ITS2 sequences of the furcocercaria differ 0.08% and 0.25%, respectively, from *C. strigeoides* and were identical to the sequences (MW244641, MW244645, MW244662) of *Cotylurus* specimens from *H. sanguisuga.*

### 3.3. Morphological Description

**Figure 3:**
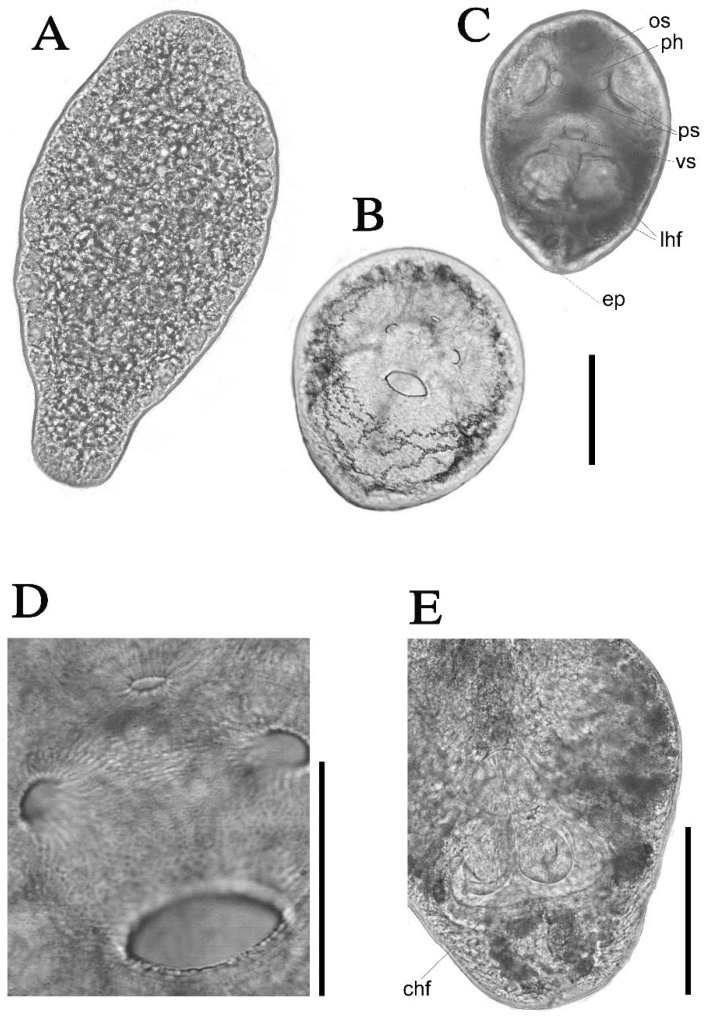
Light photomicrographs of live larval developmental stages of *Cotylurus cornutus* metacercaria. (**A**) Developing form of young metacercaria. (**B**) Cyst with granules in excretory vessels. (**C**) Cyst, ventral view. (**D**) Forebody with oral sucker in the upper part, ventral sucker in the lower part, and pseudosuckers in the lateral sides. (**E**) Holdfast organ, scale bars = 100 μm (**A**–**C**,**E**), 50 μm (**D**). Abbreviations: os, oral sucker; vs, ventral sucker; ps, pseudosuckers; ph, pharynx; ep, excretory pore; chf, cavity of holdfast organ; lhf, lobes of holdfast organ.

During dissection, developing forms of *C. cornutus* tetracotyle metacercariae (Figure 3A) were found. The young metacercariae were large in size (440–480 × 210–250 µm), with developed vacuoles, and were beginning to undergo reorganization of the body.

Voucher material: EKOI HELMI 1832 is deposited in the Helminthological Collection of the State Scientific Research Institute Nature Research Centre, Lithuania.

Measurements of tetracotyle metacercariae are based on ten cysts. Cysts were oval, slightly pear-shaped, 240–320 µm long, 170–210 µm wide. Cyst wall with two layers (an outer gelatinous hyaline layer and a tough opaque inner layer, 15–25 µm thick). There is an aperture in the cyst adjacent to the excretory pore, through which refractive granules of the excretory bladder come out. No spines on tegument. Oral sucker, 42–50 × 46–55 µm; ventral sucker 38–43 × 51–56 µm. Muscular pseudosuckers opening posterolaterally of the oral sucker. Pharynx (14–18 × 16–19 µm), short esophagus, 23–27 µm. Holdfast organ lobes behind ventral sucker. The holdfast organ of this metacercaria comprises two globular, horseshoe-shape lobes situated side-by-side at the base of the ventral depression. In some specimens, branches of the excretory system are seen due to the accumulation of refractile granules within the tubules. Refractile granules are scattered also throughout the body. In general, these morphological characteristics are consistent with older literature [38]; however, species identification in older publications, based solely on metacercarial morphology, may be misleading.

## 4. Discussion

The results of our study revealed some interesting and new scientific facts on the role of the alien snail species *L. naticoides* as an intermediate host in the life cycles of flukes and their circulation in the ecosystem. One important result in the present study is solving the problem of the taxonomic position of larvae of tetracotyle type occurring in *L. naticoides*. On the basis of the obtained DNA sequences, these metacercariae were identified as *Cotylurus cornutus* (Rudolphi 1809) Szidat, 1928. However, the taxonomic position determined are somewhat ambiguous and require more detailed discussion.

Thus, tetracotyle from *L. naticoides* fall into a well-supported major clade (Figure 1) comprising the adults and metacercariae with minimal intraspecific differences; *C. cornutus* from duck *A. platyrhynchos*, that was morphologically identified as *C. syrius* from swan *C. olor*, and larvae from different gastropod snail species. So far, metacercariae of *C. cornutus* have been recorded from a wide range of gastropod snails, mainly lymnaeid and planorbid, and leaches [38]. However, metacercariae cannot be identified to species level on the sole basis of morphology; even when the experimental infections were carried out, species identification was questionable.

The studies of Pyrka et al. [12,29] revealed two divergent phylogenetic and ecological groups within *Cotylurus*, one using leeches and the other using snails as second intermediate hosts. Based on available molecular data, *C. syrius* is associated with tetracotyles who develop in leeches, while *C. cornutus* sensu lato tetracotyle occurs exclusively in snail intermediate hosts [12]. Sequences of tetracotyles from *L. naticoides* cluster together with other sequences derived from tetracotylid metacercariae from the snail host, consistent with the established host specificity of *C. cornutus* metacercariae.

The type species of the genus *Cotylurus*, *C. cornutus*, is widely distributed in Palearctic and has been recorded in many bird species, mainly waterfowl [38]. In the mute swan, this species was reported by several authors, but most of them likely diagnosed the swan-associated species *C. syrius* individuals as *C. cornutus* [38,39]. It appears that confusion may have arisen from possible misidentifications of species of the genus *Cotylurus* based on morphology and incorrectly assigned species names to molecularly analyzed adult forms due to the variations depending on the stage or maturity of the parasites, and the hosts from which they have been isolated [39].

Pyrka et al. [12] stated that analysis of molecular data revealed three separate species-level lineages within *C. syrius* (all from *C. olor*) and four species-level lineages within *C. cornutus* (sampled from *A. platyrhynchos*). The final taxonomic hypothesis is heavily dependent on the interpretation of cox1 sequence data. The cox1 is a highly variable protein-coding gene in bilaterians and is widely used for molecular systematics of animals [40]. Variable molecular markers have the capacity to inflate species recognition, perhaps unrealistically, and as Bray et al. [41] point out, “although the data are objective, the interpretation of these data regarding species boundaries is subjective, especially when different markers conflict”. The independent source of data that molecular information brings to an investigation should be interpreted in a holistic biological and ecological context.

In our opinion, the most satisfying taxonomic hypothesis regarding delineation of species in an especially confusing strigeid genus *Cotylurus* and type species *C. cornutus* demands recognition of a complex population structure throughout its range. Regarding all available data on *C. cornutus*, we suppose that there is no significant basis for dividing genetic lineages into multiple species. Recognition of distinct species requires that molecular lineages be related to host or morphological distinctions, as in our opinion such variations are to be expected, especially in the context of molecular data from a highly variable marker such as cox1. It is more likely that this widespread species, with its wide range of definitive and intermediate hosts, has a complex population structure throughout its range, and the revealed diversification reflects interpopulation divergence.

*Cotylurus cornutus* is associated with a broad range of aquatic birds. The range of recorded intermediate hosts is also wide, which is clear evidence of euryxenicity (infection of phylogenetically unrelated species) of adult and larval stages. According to molecular data, snails of different classes (both pulmonate and prosobranch) can serve as second intermediate hosts of the tetracotyle-type metacercariae of *C. cornutus*. The basis of this exceptional pattern of host specificity is far from clear but must be partly based on the mode of transmission. These parasites spread with their definitive hosts—migratory waterfowl.

The geographical range of *C. cornutus* sensu lato is very wide. Molecular data-based tetracotyle-type metacercariae of the *C. cornutus* were found from *R. baltica* in Norway [28], with *Radix auricularia* Linnaeus, 1758 in Hokkaido, Japan [30], *R. auricularia* and *Planorbarius corneus* (Linnaeus, 1758) in Poland [12] and *R. auricularia* in China (unpubl.). It is noteworthy that isolates molecularly identical to tetracotyle from *L. naticoides* originate from Japan and China, while isolates from geographically closer regions, like Poland, have small intraspecific sequence variations which appeared random and not host- or life-stage dependent.

The comparative molecular data obtained in this research revealed that *P. fontinalis* serves as the first intermediate host of *C. strigeoides*, and this is the first record of cercariae of this species in its natural snail host. Recent molecular studies confirmed that leaches but not snails are hosts for the metacercariae of *C. strigeoides* [12,29].

The characteristics, such as morphology, physiology, behavior, diet, and life history traits, can pose very different challenges and opportunities to the parasites and may result in the parasite adapting more to one host sex than the other. Indeed, all sorts of characteristics that differ between the sexes of the host species can influence a parasite’s adaptation, as was proposed by Duneau and Ebert [42]. Regarding tetracotyle parasitism in *L. naticoides*, snail hosts become infected with active cercariae, and the level of infection may depend on what the cercariae select. As the males and females are equally abundant in population studied, chemotactic selection of host gender is most probable. On the other hand, gender-specific differences in hemocyte immuno-competence have been reported in several aquatic invertebrates. For example, studies on the immune system of the clam (*Ruditapes philippinarum* (A. Adams & Reeve, 1850)) showed that, during the pre-spawning period, females have more active hemocytes than males [43]; while in *Crassostrea hongkongensis* Lam & B. Morton, 2003 post-spawning-phase, male oysters possess a more powerful immune response than females [44]. A higher phagocytic index was observed in female triploids compared with male Pacific oysters (*C. gigas* (Thunberg, 1793) [45]. These studies suggest that gender-based differences in immune function and disease susceptibility may be responsible for differences in infection rates between the sexes.

The results of experimental investigations on behavior of *C. flabelliformis* (Faust, 1917) Van Haitsma, 1931 indicate that cercariae show host specificity and chemical gradients are used to locate slow-moving second intermediate hosts [46]. However, there are very few documented examples of parasite adaptation to host sex and very little data on differential sex-dependent infection of mollusks by digenean trematodes.

A study of impact of trematode larvae on *Viviparus viviparus* (Linnaeus, 1758) revealed sex-specific preferences for hosts and differences in the prevalence of infection between males and females; males were more heavily infected in all populations studied [15]. However, an analysis of infestation of two *Viviparus* species, *V. viviparus* and *V. contectus* (Millet, 1813), in Poland showed that the role of females in the turnover of trematode parasites is approximately three times higher than that of males [16].

## 5. Conclusions

The results of this study highlight challenges in the understanding of the biodiversity of *Cotylurus* spp., broadening knowledge about molecular diversity, taxonomy, geographic distribution, and host specificity of the parasites. Our results provide new data on the trematode fauna of *L. naticoides* and a better understanding of the role of this snail species in the circulation of the parasites in ecosystem. This study showed that the host gender is an important factor affecting the infection of *L. naticoides* with tetracotyle-type metacercariae of *C. cornutus*; males support the bulk of the parasite population.

## Figures and Tables

**Table 1:** Species subjected to molecular phylogenetic analysis with information for hosts, localities, and GenBank accession numbers.

Parasite Species	Host Species	Locality	GenBank ID * [Reference]	
			28S	5.8S-ITS2-28S
***Cotylurus cornutus*** **(mt)**	***Lithoglyphus naticoides***	**Kaunas water Reservoir, Lithuania**	**PX457620, PX457621, PX457622**	**PX457647, PX457648, PX457649, PX457650, PX457651**
***Cotylurus cornutus*** **(mt)**	***Galba truncatula***	**Gintaras Bay, Juodkrantė, Lithuania**	**PX457623**	**PX457652**
*Cotylurus cornutus* (mt)	*Radix balthica*	Lake Takvatn, Norway	KY513180 [28]	
*Cotylurus cornutus* (mt)	*Gyraulus acronicus*	Lake Takvatn, Norway	KY513181 [28]	
*Cotylurus cornutus*	*Anas platyrhynchos*	Gdańsk Pomerania, Poland	MW244637 [29]	MW244657 [29]
*Cotylurus cornutus*	*Anas platyrhynchos*	Lower Silesia, Poland	OM949854 [12]	
*Cotylurus cornutus* (mt)	*Planorbarius corneus*	Lower Silesia, Poland	OM949855 [12]	
*Cotylurus cornutus* (mt)	*Planorbarius corneus*	Lower Silesia, Poland	OM949857 [12]	
*Cotylurus cornutus* (mt)	*Planorbarius corneus*	Lower Silesia, Poland	OM949858 [12]	
*Cotylurus cornutus* (mt)	*Radix auricularia*	Lower Silesia, Poland	OM949859 [12]	
*Cotylurus cornutus* (mt)	*Lymnaea stagnalis*	Lower Silesia, Poland	OM949863 [12]	
*Cotylurus cornutus* (mt)	*Ampullaceana balthica*	Lower Silesia, Poland	OM949864 [12]	
*Cotylurus cornutus* (mt)	*Radix auricularia*	Lower Silesia, Poland	OM949865 [12]	
*Cotylurus cornutus* (mt)	*Radix auricularia*	Shihezi, Xinjiang, China	ON009068 (unpubl)	
*Cotylurus cornutus* (mt, sporocyst)	*Radix auricularia*	Hokkaido, Asahikawa, Japan	LC599502, LC599505, LC599507 [30]	
*Cotylurus cornutus* (mt)	*Radix auricularia*	Lower Silesia, Poland	OM949869 [12]	
*Cotylurus cornutus* (mt)	*Peregriana labiata (=Radix labiata)*	Lower Silesia, Poland	OM949867 [12]	
*Cotylurus cornutus* (mt)	*Lymnaea stagnalis*	Lower Silesia, Poland	OM949860 [12]	
*Cotylurus cornutus* (mt)	*Planorbarius corneus*	Lower Silesia, Poland	OM949856 [12]	
*Cotylurus cornutus (syrius)*	Cygnus olor	Lower Silesia, Poland	MW244648 [29]	MW244666 [29]
*Cotylurus gallinulae*	*Aythya affinis*	Mexico		JX977841 [31]
*Cotylurus hebraicus*	*Fulica atra*	Gdańsk Pomerania, Poland	MW244638, MW244639 [29]	MW244667, MW244668 [29]
*Cotylurus marcogliesei*	*Lophodytes cucullatus*	Hudson, Montreal area, QC, Canada	MH521248 [32]	MH521248 [32]
*Cotylurus raabei* *(=Cotylurostrigea raabei)*	*Anas platyrhynchos*	Gdańsk Pomerania	MW244649 [29]	MW244669 [29]
***Cotylurus strigeoides* (sporocyst)**	***Physa fontinalis***	**Gintaras Bay, Juodkrantė, Lithuania**	**PX457627**	**PX457707**
*Cotylurus strigeoides* (mt)	*Haemopis sanguisuga*	Lower Silesia, Poland	MW244641, MW244645 [29]	MW244662 [29]
*Cotylurus strigeoides* (mt)	*Haemopis sanguisuga*	Gdańsk Pomerania, Poland		MW244660, MW244661, [29]
*Cotylurus strigeoides* (mt)	*Haemopis sanguisuga*	Gdańsk Pomerania, Poland	MW244644 [29]	
*Cotylurus strigeoides*	*Anas platyrhynchos*	Gdańsk Pomerania, Poland	MW244640 [29]	MW244658 [29]
*Cotylurus strigeoides* (mt)	*Erpobdella octoculata*	Lower Silesia, Poland	MW244642 [29]	MW244663 [29]
*Cotylurus strigeoides*	*Anas platyrhynchos*	Poland	OM949853 [12]	
*Cotylurus syrius*	*Cygnus olor*	Gdańsk Pomerania, Poland	MW244647 [29]	MW244665 [29]
*Cotylurus* sp. (*syrius*) (mt)	*Haemopis sanguisuga*	Gdańsk Pomerania, Poland	MW244646 [29]	MW244664 [29]
*Cotylurus* sp. (cercaria, mt)	*Biomphalaria straminea*	Brazil: Minas Gerais		MN179271 MN179272 [33]
*Cotylurus* sp. (mt)	*Biomphalaria straminea*	Corrientes province, Argentina		MT276343 [34]
Outgroup				
*Alaria americana*	*Vulpes vulpes*	Canada	MH521246 [32]	MH521246 [32]
*Cardiocephaloides longicollis*	*Chroicocephalus ridibundus*	Ukraine	AY222171 [23]	MN820662 [35]
*Diplostomum ardeae*	*Nycticorax nycticorax*	Puerto Rico		MT259036 [36]
*Neodiplostomum vaucheri*	*Trachops cirrhosus*	USA	OL799108 [37]	

* Sequences generated in the present study are indicated in bold. mt—metacercaria.

**Table 2:** Prevalence of infection of *L. naticoides* by tetracotylid metacercaria according to snail sex structure.

	2010		2011		2018		2019	
Host sex	Dissected/infected	%	Dissected/infected	%	Dissected/infected	%	Dissected/infected	%
♂	61/26	42.6	120/54	45	104/32	30.8	15/11	73.3
♀	61/4	6.6	120/3	2.5	103/8	7.8	27/0	-
Not identified	169/16	9.5						
Total number of snails	291		240		207		42	

**Table 3:** Parameters of infection of *L. naticoides* with tetracotylid metacercariae.

	Year, Host Sex	Intensity of Infection (Number of Metacercariae per Specimen)												Total
		1	2	3	4	5	6	7	8	9	10	13	15	
Number of specimens infected	2010, sex not identified	4	4	3	2	1		1			1			16
	2010, ♂	12	4	2	2	2			1	1	1	1		26
	2010, ♀	2	1		1									4
	2011, ♂	12	15	8	8	3	3	1		3		1		54
	2011, ♀	2	1											3
	2018, ♂	18	6	1	4	1					1		1	32
	2018, ♀	5	2			1								8
	2019, ♂	2	5	1	2		1							11
	2019, ♀													0

## Data Availability

Newly generated rDNA sequences were deposited to NCBI GenBank (https://www.ncbi.nlm.nih.gov/nuccore) under accession numbers PX457620-PX457623, PX457627, PX457647-PX457652, PX457707, accessed on 13 October 2025. Molecular vouchers used in this study are available on request from the corresponding author.
